# Cellulose Nanocrystal Liquid Crystal Phases: Progress and Challenges in Characterization Using Rheology Coupled to Optics, Scattering, and Spectroscopy

**DOI:** 10.1021/acsnano.0c09829

**Published:** 2021-03-23

**Authors:** Roland Kádár, Stefan Spirk, Tiina Nypelö

**Affiliations:** † Department of Industrial Materials Science, Chalmers University of Technology, 412 96 Gothenburg, Sweden; ‡ Wallenberg Wood Science Center (WWSC), Chalmers University of Technology, 412 96 Gothenburg, Sweden; § Institute of Bioproducts and Paper Technology, Graz University of Technology, 8010 Graz, Austria; ∥ Department of Chemistry and Chemical Engineering, Chalmers University of Technology, 412 96 Gothenburg, Sweden

**Keywords:** cellulose, cellulose nanocrystals, alignment, assembly, flow, characterization, rheology, coupled techniques

## Abstract

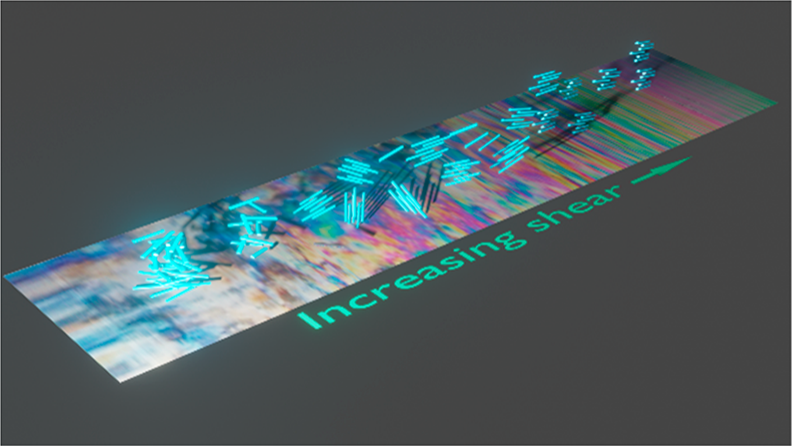

Cellulose nanocrystals (CNCs) self-assemble and can be flow-assembled to liquid crystalline orders in a water suspension. The orders range from nano- to macroscale with the contributions of individual crystals, their micron clusters, and macroscopic assemblies. The resulting hierarchies are optically active materials that exhibit iridescence, reflectance, and light transmission. Although these assemblies have the potential for future renewable materials, details about structures on different hierarchical levels that span from the nano- to the macroscale are still not unraveled. Rheological characterization is essential for investigating flow properties; however, bulk material properties make it difficult to capture the various length-scales during assembly of the suspensions, for example, in simple shear flow. Rheometry is combined with other characterization methods to allow direct analysis of the structure development in the individual hierarchical levels. While optical techniques, scattering, and spectroscopy are often used to complement rheological observations, coupling them *in situ* to allow simultaneous observation is paramount to fully understand the details of CNC assembly from liquid to solid. This Review provides an overview of achievements in the coupled analytics, as well as our current opinion about opportunities to unravel the structural distinctiveness of cellulose nanomaterials.

Cellulose nanocrystals (CNCs) are 1D nanoparticles and part of the rodlike family of lyotropic materials. Their properties differ from the micelle-forming surfactant-type lyotropic molecules. The interparticle interactions of CNCs are made complex due to the presence of the twist along the axis, which is yet to be quantified within the community. The colloidal complexity arises from the highly hydrated and chemically anisotropic surface. The anisotropy further expands to the different edges of the crystals that are seen to have more or less hydrophilic sites stemming from the equatorial and axially extended O–H and C–H functionalities. While the examples presented in this Review are on CNCs, the phenomena and principles might find use in other polysaccharide nanoparticles sharing similar surface characteristics and shape, such as chitin nanocrystals.

Self-assembly of CNCs in a water suspension into liquid crystalline or colloidal structures has inspired applications in the fields of optics and electronics.^1,2^ The rodlike nanoparticles are crystalline with a long axis that has a twist,^3^ and they assemble into liquid crystalline structures in a water suspension above critical concentration levels that depend on the dimensions and surface properties of the crystals. The liquid crystalline orders include chiral structures, the assembly of which is a function of the CNCs’ properties (*e.g.,* counterion^4^) and the media (*e.g.*,electrolyte concentration^5^ and pH value^6^).

Some details of the CNC materials are not entirely known: for example, what happens to the various structure levels during shear or drying of CNC suspensions, and how the isotropic CNC assemblies develop into liquid crystalline phases. The complexity of answering these questions in an experimental manner is evident, as the assembly involves several length-scales. The dimensions of CNCs depend on the botanical source. The typical values for wood-derived CNCs are approximately 100–300 nm in length and 3−5 nm in diameter.^140^ CNCs are produced *via* hydrolysis of dislocations of higher-hierarchy cellulose fibers, which leaves behind periodically appearing regions of higher crystallinity; hence, the individual particles are in the nanometer range. However, CNC surfaces are abundant in hydroxyl groups that lead to interparticle attraction in a water suspension *via* hydrogen bonding, and individual CNCs often form larger clusters in suspensions.

The interparticle attraction of CNCs can be reduced by introducing like-charged groups onto the particle surfaces. Sulfuric acid is a common reagent, and in addition to the hydrolysis at the dislocations, it simultaneously esterifies the cellulose nanocrystal surface, which leads to sulfate functionalities in addition to the hydroxyl groups on the surface. The sulfate half-ester groups have a negative logarithm of acid dissociation constant (p*K*_a_) that is approximately 2.^7^ This means that at pH values higher than that, the CNCs carry a net negative charge. Despite the typically low content of sulfate half-esters, the existence of the charged group makes the suspensions colloidally stable due to electrostatic repulsion. The complex interparticle interactions of the attractive hydrogen bonding and the repulsive electrostatics result in interactions between diverse length-scales of the particles and their clusters. This furthermore leads to the observation that length-scales span from the nano- to the micro- and even to the millimeter-scale, and this creates a demand for analytical devices and a selection of the length-scale of interest within those analyses.

The time-scale of self-assembly for the manufacturing of materials from CNC suspensions through drying is long, and faster processing is needed before commercial products can be feasibly manufactured. Most processing conditions require flow for transport, homogenization, and forming. In turn, flow can have a decisive influence on the structuring of the material (*e.g.*, whether or not the CNCs are aligned in the flow direction) and will therefore have a significant impact on the performance of the formed materials. For suspensions, the flow would be shear-dominated; however, extensional properties can also play a role in successful manufacturing (*e.g.*, roll application, blade coating, 3D printing, atomization, *etc*.). Applying shear to assemble dilute CNC suspensions into coatings and films^8,9^ leads to alignment in cases where the shear force is dominant over capillary forces and electrostatic interactions.^9^ Shearing CNC suspensions was also shown to improve alignment at sufficiently high shear rates.^10,11^ Compared to extensional properties, shear rheological properties are easier to determine for dispersions, and most of the scientific work on CNC dispersions—whether with the goal of understanding structuring or to determine optimal manufacturing conditions—has therefore focused on shear rheological properties and several modeling efforts have been published.^12−14^ The rheological properties of CNCs are especially important for many of the key proposed applications; among these, CNCs can serve as a viscosity modifier and stabilizer in emulsions,^15−20^ oil drilling fluids,^21−23^ inks,^24^ and food.^25^ In the case of a complex organization of CNCs, however, simultaneous characterization of the nano–micro–macro structures and the organization thereof are vital to understand the details of assembly.

## Scope and Limitations of the Present Review

**Figure 1:**
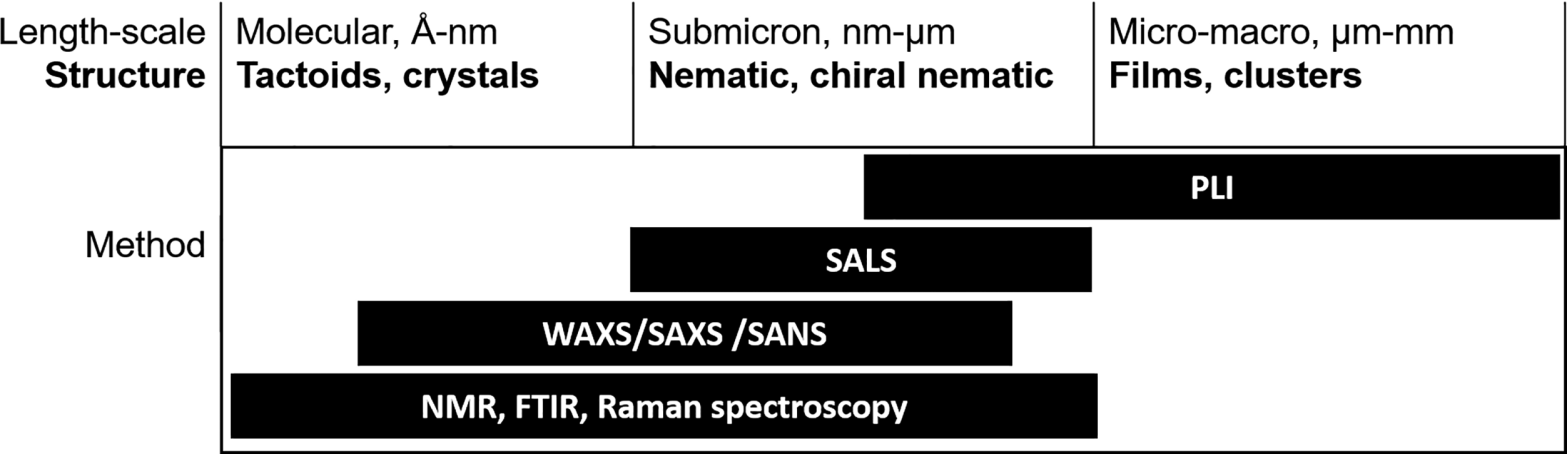
Length-scale of observations and the corresponding CNC structures, and suggested methods to be coupled to rheology to probe these structures.

This Review focuses on rheological characterization of nanocellulose suspensions in which rheology is coupled to other analytical techniques: namely, optical, scattering, and spectroscopic. The aim is to provide current opinions on the state of the art, in addition to our solutions for what is needed to answer those pending questions and lead the way to the manufacturing of CNC materials. We include hyphenated polarized light imaging (PLI), small-angle X-ray (SAXS), wide-angle X-ray (WAXS), small-angle neutron (SANS) and small-angle light scattering (SALS), nuclear magnetic resonance (NMR), Fourier transform infrared (FTIR), and Raman spectroscopy (Raman). We have summarized the reachable length-scales of the analytics and the observable phenomena in Figure 1. It should also be noted that scattering techniques (*i.e.*, SAXS and SANS) are limited to structures that are less than 120 nm in size while WAXS can be used to determine nanoscale order. Upon choosing the suitable method for addressing a research question, in our opinion, the dominating criteria are to match the length-scale of the phenomenon and the capability of the hyphenated technique.

This Review focuses on combined techniques based on rheometric instruments capable of high-precision measurements, *i.e*., commercial rotational rheometers. Therefore, this Review excludes custom-made standalone shear cells comprising numerous parallel-plate,^26−28^ cone–plate,^29^ concentric cylinders,^30−33^ and sliding plate^34−36^ variants for combining shear motion to other analytic techniques. Due to their compact design, shear cells allow superior versatility in combining with different optic, scattering, and spectroscopic techniques. As an example, for optical techniques, the compact design of shear cells means that they could be combined with advanced microscopic systems that could provide insight into morphological changes. However, shear cells generally lack the testing versatility and accuracy of commercial rotational rheometers. For both rheometer-based and shear-cell-based combined optical systems, custom variants are being developed to mitigate their drawbacks, with an increased modularity of commercial rheometers allowing for more advanced optical systems^37^ and with new measurement possibilities available on shear cells.^38^ For examples of CNC suspension structuring investigated *via* a shear flow cell coupled with polarized light imaging, see Haywood and Davis^39^ and Haywood **et al**.,^40^ while shear cells used in SAXS and SANS experiments have been reported by Eberling *et al*.^10^ and Orts *et al*.^41^

For a comprehensive understanding of rheological characterizations, we guide the reader to the following reviews on rheology of nanocellulose suspensions^42−44^ and of composites.^45^ A recent review by Fernandes *et al*.^46^ also serves as an excellent guide to the principles behind manipulating circularly polarized light with CNCs.

We will initially describe the characteristics of the structures in CNC suspensions in the Structures in CNC Suspensions and Films section, and then, we will introduce the general status of the CNC analysis in the Analysis of CNCs section. That section will also present the core of our Review, which comprises the status of rheology coupled to optical, scattering, and spectroscopy analyses. Finally, we will present our conclusions on the opportunities and challenges of a coupled analysis with CNCs in the Conclusion section.

## Structures in CNC Suspensions and Films

### Liquid Crystal Ordering

**Figure 2:**
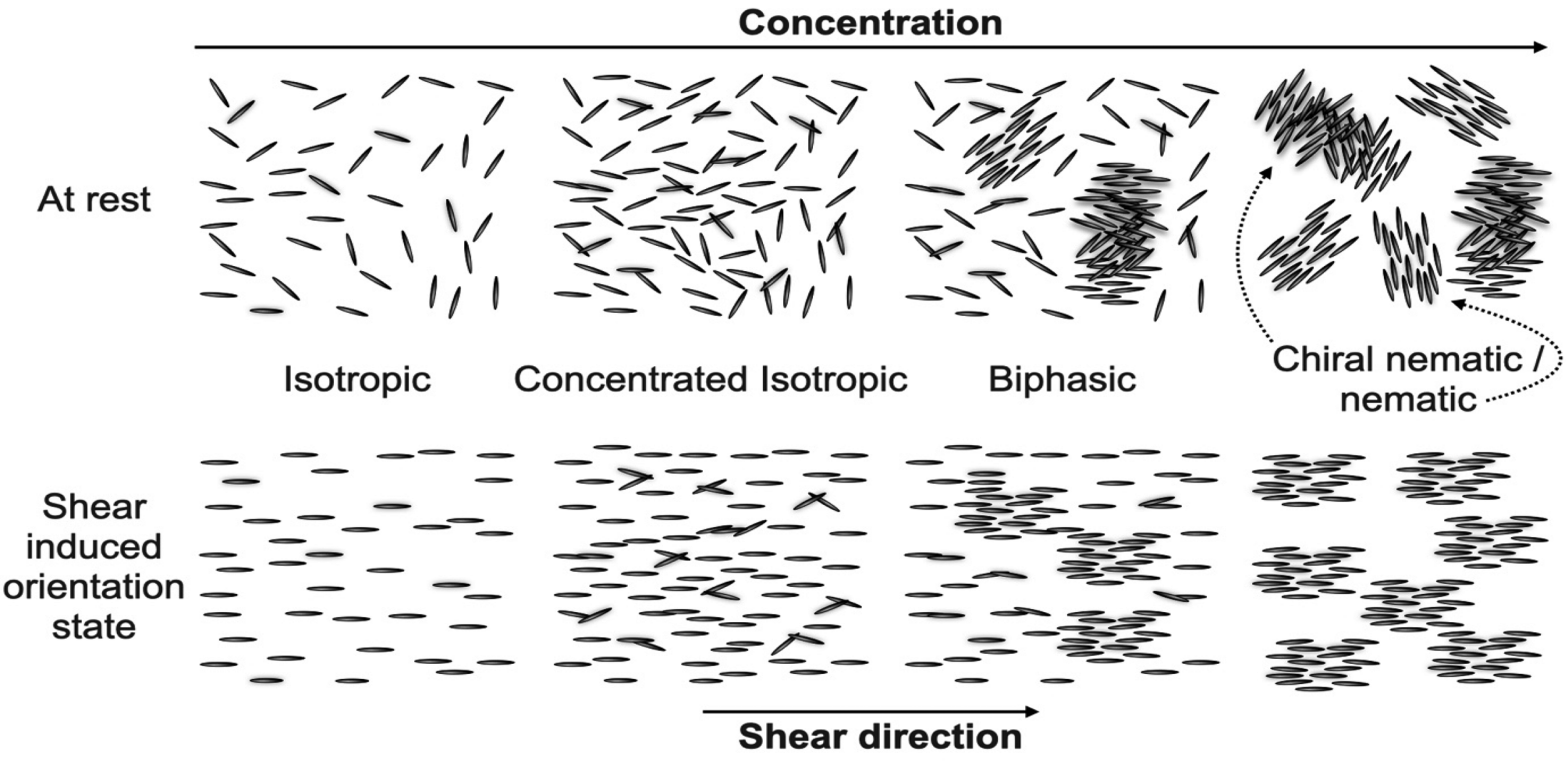
Self-assembly of CNCs in a suspension at rest with increasing concentration (upper row) and corresponding assembly in shear (bottom row).

CNCs in a dilute water suspension are reported to assemble isotropically (Figure 2). Upon an increase in concentration, the suspension shifts to semidilute and concentrated isotropic assembly. Above a critical concentration, the CNCs begin to assemble in tactoids, which are primitive phases of liquid crystalline order that later develop. The biphasic phase consists of isotropic and liquid crystalline order domains, each of which forms in equilibrium with the other. A further increase in concentration leads to a complete liquid crystalline order, and the transition from the liquid crystalline state to the gel state is an interplay between the concentration and interparticle attraction. The electrical double layer can be compressed by electrolytes and reduces the repulsion of charged particles; this reportedly triggers a transition between liquid and solid phases.^47^ The transitional structure further depends on the anisotropic–isotropic order in the CNC phase.

Honorato-Rios *et al*.^48,49^ showed that an increase in the counterion (Na^+^) concentration in a suspension of CNCs containing sulfate half-ester groups leads to loss in colloidal stability, aggregation, and percolation. The effect of the counterion, being either H^+^ or Na^+^, has been considered nondetermining since the same valence leads to equal contribution to ionic strength.^50^ In studies extending to a series of counterions, the onset of liquid crystal formation was reported to increase with the size of the counterion,^4^ and the critical aggregation concentration decreased with increasing counterion valence.^51^ The liquid crystalline order in a suspension consists of a chiral nematic phase. The nematic phase refers to orientational order for long distances, compared to the dimensions of the nanorods, and a chiral phase refers to a nematic order with a uniform twist around an axis (see also Figure 2). It has been suggested that this twist originated from the chirality of particle units;^52^ however, a comprehensive understanding of these origins is still under discussion. The total rotation of CNCs in the chiral structure is referred to as the pitch. This distance can be affected by various parameters, such as ionic strength, temperature, and suspension concentration.^5,53^ Shear-assembly of the chiral nematic phases, or even of non-nematic aggregates, leads to alignment of the CNCs toward a nematic order in which the CNCs align with the shear direction (Figure 2). This is because, at rest, the assemblies are nematic and/or chiral nematic, and the application of a shear flow leads to nematic order that is oriented to the flow direction. We note that the nematic order does not necessarily span the entire flow domain but that there are nematic domains that are separated by sizable distances, which are oriented to the flow direction (Figure 2).

The formation of the chiral liquid crystalline state occurs above a critical concentration, which depends on the CNC properties, and has been proven by polarized light microscopy and circular dichroism.^54^ The chiral phase of cellulose exhibits typically left-handed chirality. Handedness affects the optical properties of the materials: the iridescent cellulosic films prepared from CNCs reflected left-circular polarized light and transmitted right-circular polarized light when illuminated with white light,^46^ which leads to, for example, extinction of observable color when analyzed with a right-circular polarizer. Electrostatic interactions have been found to play a critical role in controlling the strength of the chirality; for example, a loss in chirality has been observed to correspond to a reduced Debye length and a mitigated Coulombic repulsion.^6^ The nematic and chiral phases in polarized light microscopy exhibit a Schlieren and fingerprint texture, respectively; in the fingerprint pattern, lines are taken as an indication of the pitch of the chiral order.

Birefringence can arise from any repeating pattern in a structure, nematic being one of them.^54^ A review by Davis^55^ described that the birefringent region of a CNC suspension decreases with decreasing concentration. Dong *et al*.^5^ found that at weight fractions below 4.6 wt % the sample was completely isotropic, and it was completely liquid crystalline above 13.1 wt %. Ureña-Benavides *et al*.^56^ found the concentration region between 3.1 and 10.4 vol % to separate into liquid crystalline and isotropic domains, and the isotropic phase disappeared at 12.1 vol %. The system was a birefringent gel between 12.6 and 14.5 vol %. Similarly, it was observed that, at high concentrations, CNC suspensions transition to the gel state where the chiral ordering is no longer detected, but instead, only birefringence appears.^57^ This is due to the kinetic arrest and the tilt of the assemblies due to crowding in the structures.

### A Little about the Rheology of CNC Suspensions

Onogi and Asada^58^ and Wissbrun^59^ reviewed the liquid crystalline polymer literature and introduced a presentation of phase transitions in flow. Both reviews schematized that the domain development in shear takes place *via* three regions in the viscosity curve. In Region I, the polydomain chiral structure remains unchanged at low shear rates, and domains are able to slide past each other; as a consequence, a first shear-thinning region is observed. Region II, a transitional region, consists of a Newtonian or nearly Newtonian region. In Region III, the second shear-thinning of the viscosity curve materializes, and it has been suggested that the general alignment of the liquid crystal polymer occurs here. We note here that the flow complexity poses significant challenges to their theoretical understanding,^60^ especially when considering the normal stress differences and transient behavior of liquid crystalline polymers.^61−63^ Much like in liquid crystalline polymers, where the three-region behavior has been shown to be a persistent but not universal feature of the viscosity curves, depending on their type and experimental conditions,^62,64^ experimental and numerical studies have generally attributed it to the liquid crystalline phase,^64−66^ elucidating the three-region behavior in CNC suspensions remains a substantial challenge. The scientific literature contains a significant number of results showcasing the three-region steady shear viscosity behavior in self-assembling CNC suspensions.^40,41,56,57,67−75^ However, there is no general consensus in assigning the behavior to a particular phase. For example, the results of Ureña-Benavides *et al*.^56^ and Kádár *et al*.^74^ showed the three-region behavior to correspond to the biphasic phase. In contrast, Orts *et al*.^41^ have identified the behavior for concentrations corresponding to the liquid crystalline phase. Furthermore, Shafiei-Sabet *et al*.^57^ and Haywood *et al*.^40^ have observed the three-region behavior for concentrations corresponding to both biphasic and liquid crystalline phases. In particular, Haywood *et al*. have shown that while the three-region behavior could be relatively clearly observed for biphasic concentrations, by fitting different regions of the viscosity curve with the power law model on viscosity curves otherwise appearing to show only one shear-thinning region, the three-region behavior could be identified over a broader range of concentrations.^40^ Several aspects could be responsible for the variety of the results reported, including the differing methods for identifying the CNC phases and even difficulties in clearly identifying the three regions in the viscosity.^76^ Perhaps most relevant is the sensitivity of CNC rheological behavior to the sample preparation procedure. Regarding the latter, Shafiei-Sabet *et al*.^57^ have shown that for a fixed CNC concentration the viscosity functions can vary from having one apparent single shear-thinning region for unsonicated suspensions to a three-region behavior with increasing the sonication. Buffa *et al*.^73^ also showed that unmodified CNC suspensions in water exhibit the shear-thinning regions of different slopes that are associated with structural breakage and an alignment of domains and CNCs. An investigation using gum arabic or CNC surface modifications further elucidated that rheology is sensitive to surface modifications and the CNCs’ matrices. CNCs with the gum additive or with hydroxyl and carboxyl functionality were stable in a suspension and exhibited concentration-dependent organization into isotropic and anisotropic phases. However, the silanized CNCs only organized in flow. A decrease in the steady-shear oscillation was observed when the CNC surface properties were changed *via* oxidation (*i.e.*, TEMPO) or silanization.

The liquid crystal structure of CNCs can be modulated using specific ionic effects.^47^ The weakly hydrated SCN^–^ ion enhances CNC colloidal stability but weakens gel properties.^47^ Formation of the liquid crystal hydroglass is the result of the arrangement of the CNCs and the gelation thereof. In an investigation of suspensions that ranged from 1 to 11.9 wt %, four phases were identified in the CNC suspensions: liquid, viscoelastic, repulsive soft glass, and attractive glass/gel. An increase in ionic strength results in a decrease in Debye length, which in turn leads to CNCs accessing one another in closer proximity. An increase in the concentration of the charged CNCs generates a transformation from a viscous liquid to a rheological solid phase, which is referred to as repulsive glass.^77^ In the soft glass state, the particle dynamics are arrested, and they exhibit a yield stress and storage modulus that is larger than the loss modulus. When a shear flow is applied to this system, rearrangement occurs for shear stresses above the yield stress of the network. Shear can lead to a progressive decrease in hydrogen bonding density that cannot be recovered after the flow due to the repulsion that dominates the restructuring.^78^

Li *et al*.^6^ found that the electrostatic forces between rodlike polyelectrolytes promote a perpendicular orientation. The expected phase behavior is characterized by the transition between the nematic and chiral nematic phases and is primarily dictated by the twist parameter, which is the ratio of the Debye length to the effective diameter of the rod. Buffa *et al*.^73^ reported a change to the particle aspect ratio in a suspension; this was determined *via* a change in the effective diameter (*i.e.*, the space occupied in the suspension and originating from the colloidal properties), which was also an indication that the rods associate in flow.

## Analysis of CNCs

### Status of Standardization

Because nanomaterials might have different properties than their macroscopic counterparts, there have been past efforts to standardize the terms related to nanomaterials based on cellulose.^79,80^ These studies include the definition and forms of nanocellulose and propose standardized methods to analyze materials.^81^ In terms of CNCs, several standards have been published that contain procedures for sample preparation (*i.e.*, dispersion), as well as characterization and ways to interpret the data.^82,83^ Moreover, there are several initiatives that are expected to further improve and standardize cellulose nanomaterials;^84−87^ these include characterizing individualized cellulose nanofibrils, studying particle size distributions for CNCs, examining the crystallinity of cellulose nanomaterials by powder X-ray diffraction, and determining CNC sulfur and sulfate half-ester content, to name a few. The development of standardized procedures for CNC preparation, dispersion, and characterization is one of the key challenges to accomplish to make results better comparable between different laboratories.

Although rheology is an important parameter for the characterization of CNC suspensions, to the best of our knowledge, rheological experiments have not been considered in such standardization efforts. However, rheology is a widely used method to assess polymer properties, and as such, we view consideration of it in future standardization efforts to be crucial.

### Rheology Coupled to Other Analytical Techniques

This section represents the core of this Review and introduces the state of the art of rheo-optics, rheo-scattering, and rheo-spectroscopy for analysis of CNC suspensions.

### Rheo-Optics

**Figure 3:**
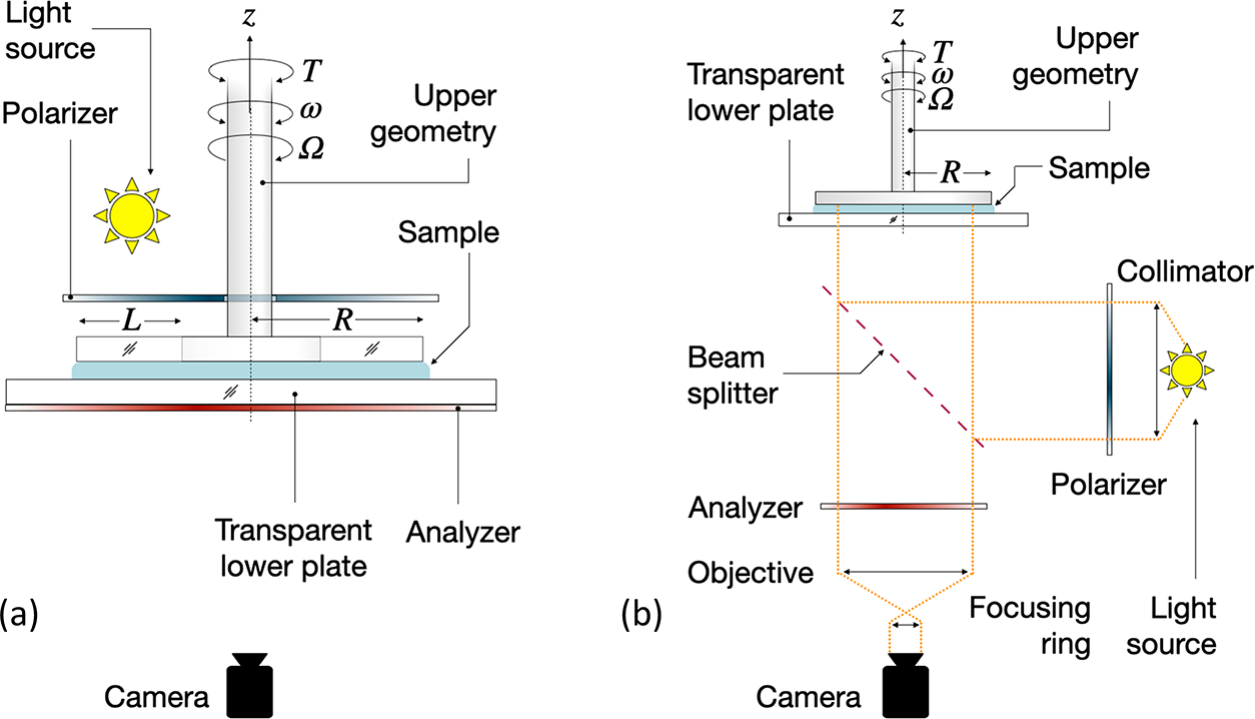
General outline of typical rheo-PLI configurations in (a) transmission^74^ and (b) reflection^88^ modes. The schematics are based on the cited publications, and more details can be found therein. Note that several setup configurations are possible depending on the design.

**Figure 4:**
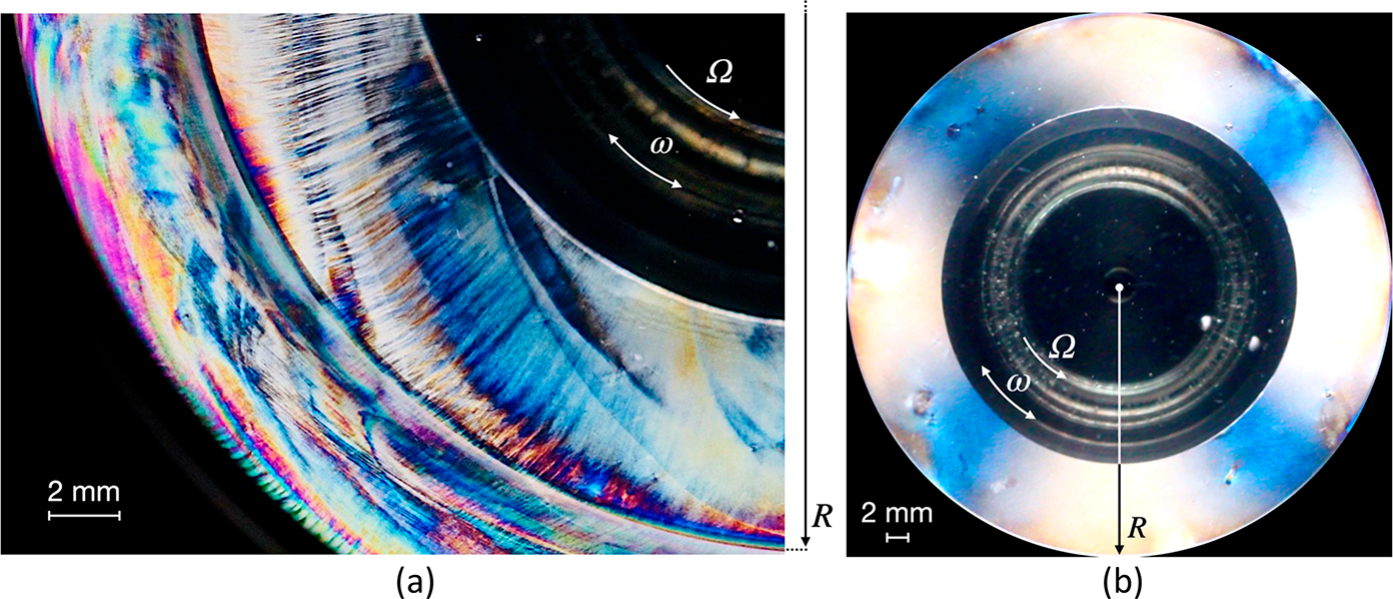
(a) CNC birefringence patterns (unpublished figure provided by R.K.) visualized using a custom transmission mode setup similar to that reported elsewhere.^74^ The radius *R* marks the edge of the measuring plate radius (2*R* = 43 mm). The visualization was created immediately after the sample was squeezed into the measuring gap. (b) Maltese-cross pattern visualized at high shear rates through the same setup. Credit: photo courtesy of M. Fazilati; used with permission.

Combining rheology with optical visualization is an established method for investigating the dynamics of complex fluids.^89,90^ The associated hyphenated techniques are of significant importance for complex fluids, because they have the potential to relate bulk-averaged rheological properties to microstructural dynamics using accessible means in terms of incident radiation (*i.e.*, visible light).^89^ Therefore, they are easy to implement into commercial rotational rheometers. For CNC suspensions, a cross-polarized setup, in which the sample is placed between two linear polarizers that are rotated 90° relative to each other, is one of the most used configurations for polarized light imaging (PLI). Several publications that describe in detail recent advances in rheo-PLI are available.^88,91,92^ Rheo-PLI investigations on CNC dispersions have been performed using a variety of PLI setups, including in transmission mode^57,68,69,74^ and in reflection mode *via* a collimator-beam splitter setup (Figure 3).^19,88^ In the event of the latter, regular steel moving geometries can be used, as opposed to the former, for which a special transparent moving geometry that limits the visualization area is needed.^74^ A disadvantage of rheo-PLI in reflection mode is that a steel geometry background may not always provide an ideal contrast for observing nematogenic structures. The PLI observation length-scale can also vary according to the type of camera objective used. For a microscopic lens, the observation length-scale varied between ∼50 and 250 μm^57,68,69^ and enabled the direct identification of mesoscale chiral nematic structures; for a macroscopic lens, the observation length-scale was varied from *d*, where *d* is the diameter of the measuring geometry,^88^ to a quadrant thereof.^74^ The orientation of particles in the flow direction can thus be readily identified based on the onset of a “Maltese-cross” pattern,^92^ at the expense of losing the ability to directly observe individual mesoscopic structures (see also Figure 4).

Early rheo-PLI experiments were performed on nanocellulose whiskers—with a length in the range of micrometers—by utilizing a custom transparent cone–plate geometry.^67,93^ The authors of the studies reported an absence of equidistant striations that are characteristic of chiral liquid crystals for 3 wt % whisker dispersions at rest.^67^ The apparent disparity between the relatively high concentration and the absence of identifiable chiral liquid crystals was explained by either the chiral domains or the chiral pitch being too small to be observed. For concentrated suspensions (>2 wt %), a shear-induced “banded” periodic morphology was observed in the PLI visualizations, with the “bands” oriented 45° relative to the shear direction. The critical shear rate for the onset of the banded morphology was estimated to be approximately 1 s^–1^.^67^

More than a decade later, Shafiei-Sabet *et al*. renewed interest in rheo-PLI and CNCs using an improved plate-to-plate visualization setup and a PLI-observation length-scale of approximately 50–250 μm. Thus, a fingerprint texture for dispersions of CNCs at different concentrations in the liquid crystalline phase and at different degrees of sulfation—4.39 and 3.55 OSO_3_H/100 anhydroglucose units, respectively—could be observed.^57,68^ The fingerprint structures for the biphasic samples significantly increased in size with increasing concentration. While the transition to the gel state displayed birefringence, this occurred without the fingerprint texture. A lower degree of sulfation results in decreased intermolecular interactions, which results in gel-phase formation at a lower concentration.^68^ The authors associated the fingerprint texture to chiral nematic domains at rest, and it could also be correlated to the sonication level of the dispersions at rest. Unsonicated samples showed birefringence patterns with an absence of the fingerprint textures and increasingly pronounced textures with increasing sonication levels.^68^ Increasing sonication also resulted in an expansion of the samples and an increase in pitch due to expansion of the electrical double layer;^57^ see also Beck *et al*.^94^ The transition from liquid crystalline concentrations to the gel state can therefore be determined by the absence of fingerprints with increasing CNC concentrations.^68^ At a constant shear rate under the influence of temperature, fingerprint textures were shown to diminish with increasing temperatures, and this trend was reflected in the respective viscosity functions thereof.^57^

Irrespective of the starting mesophase, the influence of increasing shear-rates in sufficiently nonlinear conditions follows a similar sequence. Orientation in the flow direction of the mesophase could be observed around 0.1 s^–1^ in the form of textured domains that increasingly stretch and orient in the flow direction. Along with increasing shear-rates, the PLI micrographs become increasingly darker (*i.e.*, light intensity). Here, a distinction can be made between sonicated and unsonicated dispersions, in that for shear rates over 0.5 s^–1^ the sonicated samples became darker, compared to the unsonicated samples at the same concentration.^57^ The micrographs were correlated to the viscosity functions of the CNC dispersions. Concentrations showing the three-region viscosity curves were characterized by fingerprint textures that were present at rest within the chiral nematic domains. These became progressively distorted, followed by orientation in the flow direction and increasingly dark coloration, the latter being assigned to the breakup of nematic structures and orientation of the primary CNC particles in the flow direction.^68^ For a collection of CNC dispersions with increasing ζ potential due to the addition of NaCl, chiral nematic domains, which were estimated to be approximately 100 μm in size in the absence of NaCl, significantly decreased in size until they disappeared above 10 mM NaCl.^69^ The aging and yielding of CNC dispersions were investigated by combining the previously used rheo-PLI setup with light-scattering-echo (LS-echo) measurements.^95^ Dynamic oscillatory tests revealed that irreversible changes in the particle positions could be recorded past the yield strain, which were attributed to the strain at which storage and loss moduli cross over.^95^

The orientation dynamics of CNC dispersions for 3D printing applications was investigated by Hausmann *et al*.^19^ An important feature of the rheo-PLI setup used in this study is that the visualization length-scale includes the full radius of the geometry, and thus, the distribution of local shear rates with the radial coordinate can be exploited.^91^ It is worth noting that this study was performed on a parallel-plate setup with a gap of 0.2 mm and that the results were validated for higher gaps and even a cone–plate geometry with minimal differences found between the measurements.^19^ For the high CNC concentrations (*i.e.*, up to 40 wt %) investigated, the conditions for particle orientation in simple shear flow relied on inducing stresses above the yield stress of the sample; whereafter the CNC orientation dynamics in the flow direction were determined according to the applied shear rate and specific CNC network interactions.^19^ At rest and at low shear rates, a significant increase in the nematic domains was observed with increasing CNC concentrations. The alignment of CNC in the flow direction could be readily observed through the appearance of the Maltese-cross pattern, which was easily observable in the reflection mode of the rheo-PLI setup.^91,92^ The authors also applied their PLI observations to 3D printing extrusion flows, where the nonlinear velocity gradient results in a distribution of shear rates within the radius of the extrusion die.

The influence of CNC concentration on flow-induced birefringence patterns was also investigated using a macroscopic lens setup with PLI in transmission mode.^74^ A notable improvement in this custom setup was the addition of a white background, which enhanced the contrasting birefringence patterns, resulting in vibrant color patterns (Figure 4a). The authors of this study performed visualizations also during oscillatory shear-strain amplitude sweeps, and long recordings were presented in the form of space–time diagrams. They identified the critical transition shear rates for linear (*i.e.*, no visible birefringence pattern distortions), nonlinear (*i.e.*, pattern distortions), and the onset and development of a uniformly oriented flow field (*i.e.*, the Maltese-cross pattern) (Figure 4b). The presence of coloration in the Maltese-cross patterns suggested that it was possible for nematic domains to survive the orientation in the flow direction, which would contradict some of the previous observations.^19,68,74^ Interestingly, the general orientation in the flow direction identified from birefringence pattern dynamics was difficult to detect in the corresponding shear viscosity functions. Furthermore, a transition state was revealed prior to the onset of a uniform orientation in the flow direction, which consisted of radially periodic birefringence patterns at intermediate shear rates (*i.e.*, the Maltese-cross pattern). A markedly different feature of the radially periodic birefringence patterns was that they were stable with respect to the change in shear direction in the oscillatory flow. These radially periodic birefringence patterns corresponded to the organization of biphasic CNC suspensions in flow as an intermediate state as they transitioned toward alignment.^74^

### Rheo-Scattering

**Figure 5:**
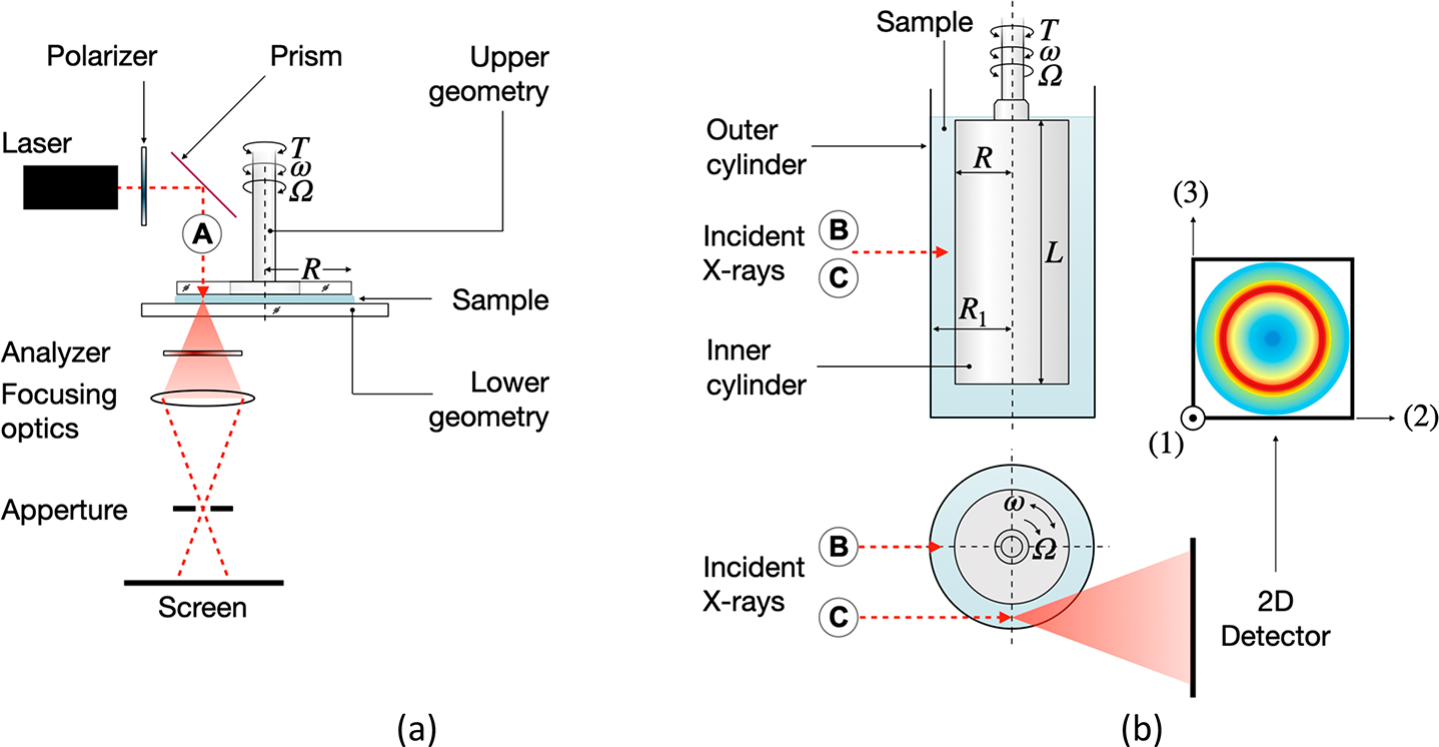
Generic illustrations of (a) rheo-SALS^97^ and (b) rheo-SAXS/WAXS/SANS setups.^96^ Specific details such as beam stops and differences in detector positioning between SAXS and WAXS have been omitted. Note that several construction configurations are possible depending on the design. (1–3) represent the velocity, velocity gradient, and vorticity directions, respectively. Labels A–C correspond to 1–3, plane (parallel-plate), radial, and tangential and incident beam configurations. The illustrated scattering pattern in part b corresponds to random sample orientation.

SAXS, WAXS, SANS, and SALS can be combined with rheological characterizations of CNC suspensions. Generic outlines of rheo-SALS and rheo-X-ray scattering setups are presented in Figure 5. We note that the incident beam configurations A–C are possible for both setups in Figure 5a,b.^96^ In addition, while the SALS setup was detailed for a plate–plate geometry and the X-ray/neutron scattering setup for a concentric cylinder measuring geometries, the two are interchangeable. Finally, we add that the materials used in the manufacturing of measuring geometries vary according to whether X-rays or neutrons are used. The status and benefits for these analyses of cellulose nanocrystal suspensions will be discussed in this section.

#### Rheo-SAXS, Rheo-SANS, and Rheo-WAXS

Both SAXS and SANS are capable of probing the nanometer length-scale. Smaller particles scatter at larger angles, and those angles are converted to *q*-values that can be used to characterize dimensions in space (*D* = 2π/*q*). Analyses conducted at low *q*-values reach particle interactions, and those at high *q*-values reach the particle surface. In a suspension, a shift to higher *q*-values occurs when the suspensions become more concentrated, and the particles come close to each other. Rheology characterizes several length-scales at the same time, and hence, combining the scattering analysis with the rheological analysis enables an extension of the length-scale of both techniques. In comparison to the PLI techniques presented in the previous section, scattering techniques can probe shorter length-scales.

In SAXS, X-rays interact with electrons in the shells around the nuclei. As the interaction scales with the amount of electrons, elements with higher atomic number provide more intensity in SAXS experiments than those with lower. The scattering pattern is typically radially integrated into a diffractogram that presents the levels of intensity over the *q*-ranges, within which the correlation peak region can be used to probe interactions between the particles and the form-factor region offers an indication of the particle shape and orientation.^98^ Utilizing azimuthal integration instead of radial integration allows analysis of the orientation.^99^ The alignment analysis has been applied to reveal the alignment of crystalline cellulose in suspensions using a synchrotron source.^10^ The suspension at rest exhibited random orientation, low shear-rate orientation in the plane that is perpendicular to the shear direction, and high shear rates (5 s^–1^ and higher) in the horizontal alignment in the shear direction; the difference between alignment that is perpendicular to or in the shear direction was determined to be due to the fact that domains of interacting particles align at low shear rates, while individual particles align at high shear rates. Rey *et al*.^100^ employed a SAXS orientation analysis coupled to cross-flow ultrafiltration and combined this with rheometry; a separate rheology characterization was used for calculation of the shear stress inside the accumulated layers to reveal details related to layer formation and flow. Despite the potential of directly coupling rheology and SAXS (rheo-SAXS) in CNC analysis, to the best of our knowledge, only limited oral communications are available.^101^ The wide-angle region of the X-ray scattering has also been applied to study the real-time orientation of CNCs in shear flow;^102^ it was noted that the morphology of the CNC films was affected by shear velocity, the concentration of the precursor suspension, and the evaporation temperature.

In SANS, neutrons interact with nuclei, and the contrast depends on atomic masses, which allows for an isotope-specific analysis. For water-based systems, the SANS is typically performed in D_2_O, because deuterium scatters more than hydrogen. Recently, van Rie *et al*.^103^ utilized SANS to investigate the alignment of gold nanorods in a chiral CNC suspension. The ratio of SANS intensity to the *q*-value curves of the suspensions with 5 and 8 wt % CNC concentration (without gold rods) displayed distinct maxima that were associated with the cholesteric liquid crystal phase, and an increase in the CNC concentration shifted the position of these maxima to higher *q*-values, which indicated a decrease in CNC distance from 53 to 43 nm with a 5–8 wt % suspension. Haywood *et al*.^40^ used rheo-SANS for CNC suspension characterization: tests were performed at 10 °C to avoid evaporation, using a titanium cup and hollow bob geometry with SANS measurements performed in radial and tangential configurations (see Figure 5B,C). The low-concentration suspensions (2.49 and 3.16 vol %) exhibited ring diffraction patterns and order parameters expected for Newtonian fluid behavior.^40^ In contrast, both biphasic (3.83–5.83 vol %) and liquid crystalline (6.50–8.48 vol %) concentrations exhibited a three-region behavior. This was observed in both the steady shear viscosity functions and order parameter dependence on the shear rate. However, while the three-region behavior was difficult to identify in the viscosity functions, especially in the liquid crystalline concentration range, the order parameter showed well-defined regions. For the biphasic 4.5 vol % sample, anisotropy was seen to increase with increasing shear rate in Region I. In Region II, the anisotropy remained relatively constant, with an isotropic ring still visible. This suggested that while a portion of the rods aligned in the flow direction, significant portions of the sample remained randomly oriented. The anisotropic intensity was significantly higher at the higher end of applied shear rates (Region III), suggesting a predominant orientation in the flow direction. Samples exhibiting a rheological gel behavior were affected by slow relaxation kinetics in the first region with respect to the deformation history prior to the beginning of the tests and with increasing CNC concentration required higher shear rates to exhibit significant anisotropy, as expected. While the study provided a very intriguing array of insights into the phase behavior dynamics, the authors note that further investigation would be needed to fully understand the three-region behavior in CNC suspensions.^40^ We also note that Haywood *et al*.^40^ resorted to polarized light optical visualizations on a shear cell to better visualize the structural changes induced by the flow at the macroscale. This emphasizes the need for complementarity even between different combined rheological techniques to access the multiple structural length-scales.

An issue that is relevant in general for rheo-SANS (and rheo-NMR, see the next section) is how the presence of deuterated solvents impacts phase stability and even formation of liquid crystalline phases. Despite being chemically identical, there are differences in the physical properties of H_2_O and D_2_O. Many of the differences originate from a distinctly different hydrogen bonding behavior. While the length of hydrogen bonds is more or less the same, the strength of H-bonds in D_2_O is 5–6% higher than in H_2_O.^104^ While this phenomenon has been addressed for numerous materials forming liquid crystalline phases,^105−107^ for CNCs this is hardly reported. It is, however, clear that the presence of D_2_O will affect the formation and stability of liquid crystalline phases as the hydrogen bond energy is definitely an important thermodynamic factor in the formation of such phases. Furthermore, if the pH value is changed (*e.g.*, by introduction of NaOD), this may lead to deviation in the formation of such liquid crystalline states. Research in this area is definitely needed to evaluate the isotope effect on cellulose liquid crystalline phases.

#### Rheo-SALS

Rheology and SALS data have been collected simultaneously using a commercially available SALS attached to a rheometer,^108^ and the results revealed that increasing orientation had a limit when it came to the concentration level, as high concentrations hindered this organization. This was determined to be due to two facts: while the high shear force initially disintegrated the entangled CNCs, an additional increase in CNC concentration led to densification of the aggregates, and as the physical constraints became stronger, the structures were able to withstand the shear stresses. Light scattering has also been coupled to rheology and combined with optical polarized microscopy investigation on CNCs in an LS-echo setup.^109^ There the conventional rheometry is combined with dynamic light scattering (DLS) techniques under oscillatory shear that allows measurement of the autocorrelation function of the scattering intensity of samples subjected to oscillatory shear deformation. They present that the particle motions in the below-yield strain are reversible, while they are irreversible above the arrangements.

### Rheo-Spectroscopy

#### Rheo-NMR Spectroscopy

The underlying principle of nuclear magnetic resonance (NMR) spectroscopy is to excite nuclei with a spin ≠ 0. Commonly employed nuclei include ^1^H, ^13^C, ^15^N, ^19^F, ^29^Si, ^31^P, and ^77^Se; the shared feature of these nuclei is that they have a spin of 1/2, which results in well-resolved spectra, while quadrupolar nuclei (*I* > 1/2) usually result in signals with significant peak broadening, unless there is symmetry around the nucleus.^110^ Nevertheless, ^2^H (*I* = 1) is particularly relevant when combining NMR spectroscopy and rheology.

The chemical environment of the nucleus in a molecule can be easily assessed by an analysis of the coupling patterns and coupling constants to other nuclei in combination with their chemical shift in relation to the frequency of a standard, mostly tetramethylsilane. In other words, the different responses are due to excitation caused by the chemical functionalities inside the molecule. Multidimensional correlation experiments are typically performed to resolve the molecular structure of small organic and large polymeric molecules such as proteins by correlating the chemical shifts and coupling constants.

An NMR experiment is designed in such a way that a high-frequency pulse is used to excite the molecule, upon which a decay signal (FID) is detected, followed by a waiting time (*i.e.*, a delay time) that is required to achieve relaxation of the system into the ground state, before the next pulse is applied.^110^ The phenomenon that can be exploited for rheology measurements is that both the chemical shift and the relaxation were dependent on the size, orientation, and mobility of the molecules in a solution. Apart from chemical functionality, relaxation times depend on the nucleus and the resonance frequency thereof. For ^1^H, which is the most commonly employed nucleus, relaxation times can range from a few microseconds for small molecules in nonviscous solutions to several seconds in polymeric solutions with high viscosity.

There is a significant overlap between the available time-scales in rheology and NMR spectroscopy, which allows different types of phenomena to be studied;^111^ for example, complex fluids often feature nonlinear rheological behavior, which is a function of evolving time. In the following, we will briefly elaborate on different setups that were able to connect rheology with information gained from NMR spectroscopy. Martins *et al*.^112^ used NMR spectroscopy to extract rheological data. Experiments that were denoted as “rheo-NMR” were later developed by Nakatani *et al*.,^113^ who explored sheared polymer melts using *in situ* NMR studies; this study took up the work developed by Vera and Grutzner^114,115^ and Lacelle *et al*.^116^

**Figure 6:**
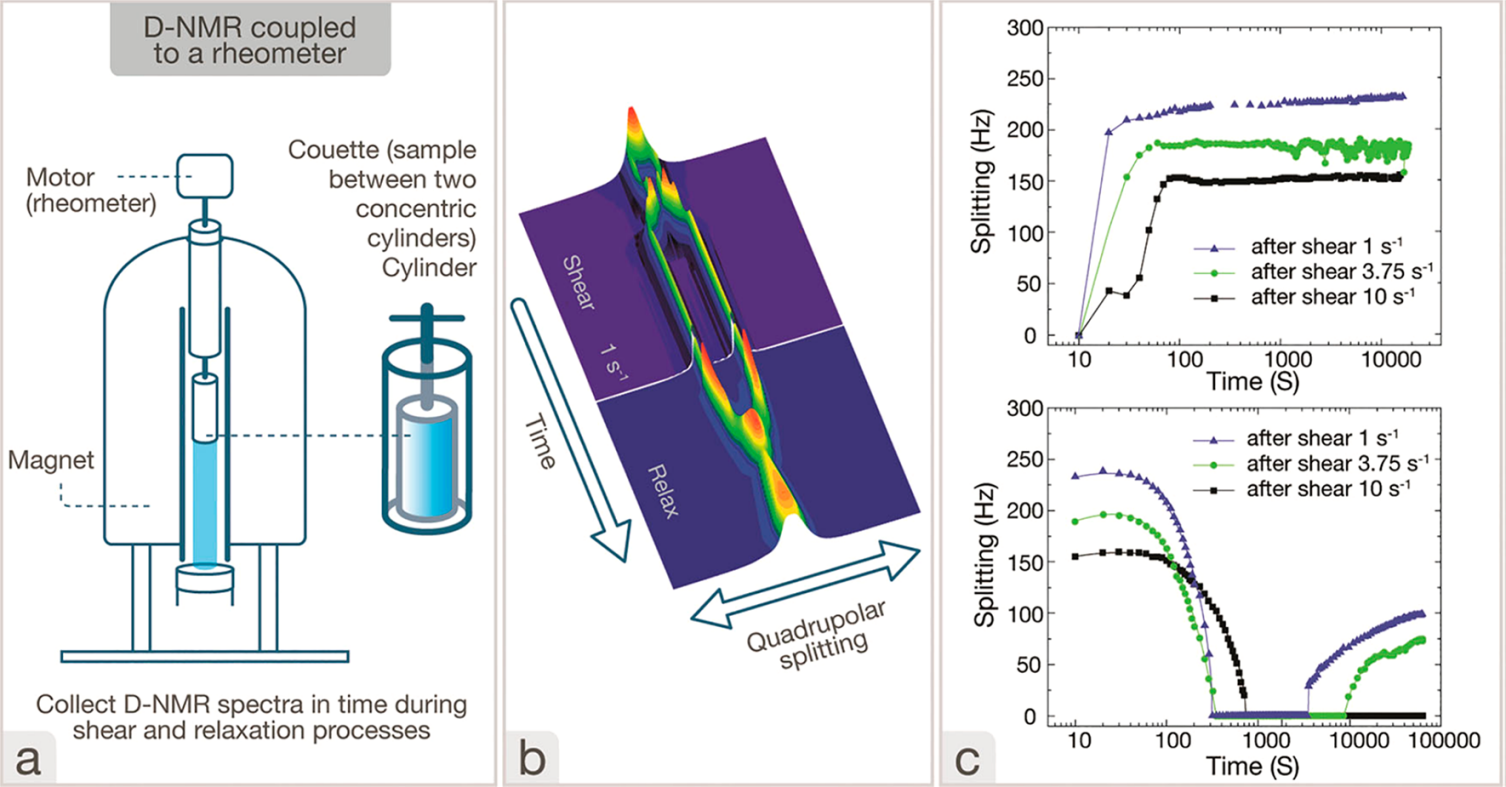
(a) Description of rheo-NMR technique and the Taylor–Couette flow cell: (1) Couette (sample between two concentric cylinders) cylinder. (2) Motor (rheometer). (3) Magnet. (4) Collect ^2^H–NMR spectra in time during the shear and relaxation processes. (b) Representative graph corresponding to a collection of deuterium NMR spectra with time for an LC–HPC/D_2_O (50 wt %) during shearing at 1 s^–1^ and after cessation of shear during the relaxation process. (c) Evolution of the quadrupolar peak splitting as a function of time during shear (top) (1, 3.75, and 10 s^–1^) and the further relaxation process (bottom) for LC–HPC/D_2_O (50% w/w). Reprinted with permission from ref (119). Copyright 2017 Taylor & Francis Ltd.

In rheo-NMR, two main concepts predominate. The first refers to velocity imaging (*i.e.*, the flow field that will be spatially mapped and analyzed). This approach is often termed “dynamic NMR microscopy”, and it has been the subject of several reviews;^117,118^ it has not yet been applied to cellulose nanocrystals and will therefore not be discussed further. The second concept does not resolve spatial information, but it is designed according to the behavior of a sample under flow conditions. In this setup, NMR spectroscopy can be employed to determine the orientation of the liquid crystal phase in flow. The relaxation of the director—from an out-of-equilibrium orientation back toward the magnetic field or the director orientation under continuous rotation—can be exploited to assess such rheological properties as viscosity and the elastic constants of nematic phases (Figure 6).^119^ For more details on the experimental specifics, we refer to the thorough account given by Schmidt.^111^

For CNCs, the literature on rheo-NMR is scarce. Echeverria *et al*.^120^ published an account of the effect of CNCs in LC–HPC (hydroxypropyl cellulose) at low shear rates. They acquired deuterium spectra and the corresponding *T*_2_-values and were able to assess that even small amounts of CNCs affected the mobility of LC–HPC chains. They concluded that the flow of the LC–HPC chiral polydomains over each other could be impeded by the presence of the CNCs in solution. The effect scales with CNC concentration and at 2 wt % resulted in an achieved degree of order that was recovered more quickly, compared to pure LC–HPC and LC–HPC with 0.1 and 1 wt % CNCs. Furthermore, additional strain units were required to induce some degree of order for the 2 wt % CNC sample. The authors speculated that the presence of 2 wt % CNCs affected the first shear-thinning regime of the typical three-region curve (see the discussion in previous sections). In the case of CNCs, it has been suggested that the dynamics of chiral domains that flow over each other are compromised under high shear conditions. It must be mentioned that the CNCs did not affect the chiral structure of the LC–HPC/water (50 wt %) mixture, which was proven by polarized optical microscopy. The authors also showed that the shear dynamic moduli, *G*′ and *G*″, increased with increasing CNC content.^120^

Another work by the same authors focused on the effect of higher shear rates on the behavior of LC–HPC–CNC dispersions.^121^ Similar to the previous report, LC–HPC–CNC dispersions with different CNC content (*i.e.*, 1, 2, and 5 wt %) were prepared. Liquid-like behavior (*i.e.*, *G*′ < *G*″) was observed for the lower CNC concentrations, while solid dispersion-like behavior (*i.e.*, *G*′ > *G*″; 5 wt % CNCs) was identified for higher concentrations.^121^ An analysis of the viscoelastic properties demonstrated that CNCs reinforce the solution as both *G*′ and *G*′′ increased. Incorporating 5 wt % CNCs into the LC–HPC yields a change from liquid-like behavior to gel-like behavior. Rheo-NMR and dynamic measurements showed that, after cessation of the shear, the presence of CNCs in the HPC chains restricts the mobility of LC–HPC chains; this confined mobility leads to a slow recovery of the chiral structure at low concentrations (1 and 2 wt %), while this formation does not occur at all at 5 wt % CNCs. This NMR study provided evidence for why CNCs already act as reinforcing agents in LC phases and proved that there is a concentration limit upon which the LC nature of the matrix will be destroyed.

#### Rheo-FTIR and Rheo-Raman Spectroscopy

Infrared (IR) and Raman spectroscopy rely on the identification of vibrational modes within molecules and are widely employed in the structural identification of small and large molecules. IR spectroscopy is capable of detecting vibrations when the dipole moment is changed during IR absorption; a permanent dipole is not required. Vibrations around centers of symmetry are usually not IR-active, but they are often Raman-active.^122^ In Raman spectroscopy, an inelastic scattering of photons that is the result of interactions of vibrational modes in molecules is exploited; this scattering causes a shift in the energy of the employed light source—typically a laser—resulting in a so-called Raman shift. Molecules must have polarizable dipoles to fulfill this rule, since Raman intensity correlates with the polarizability change during interactions with the laser.^122^

**Figure 7:**
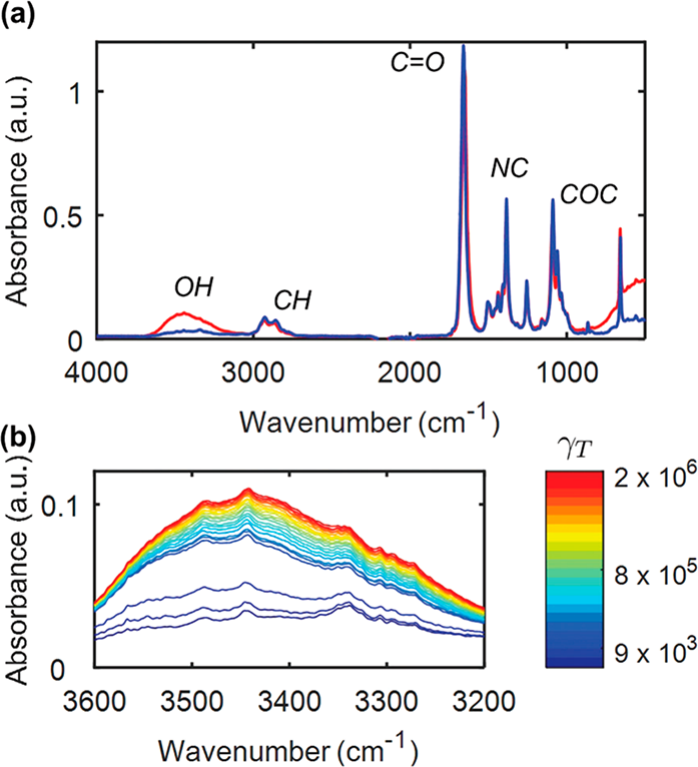
Rheo-FTIR of CNC–polymer gels: (a) Mid-IR spectrum of a UV-curable gel with 14% CNCs, acquired from the sample at rest prior to any shear (blue line) and after a large total strain (γ_T_ ≈ 10^6^) accumulated during a creep experiment at *s* = 105 Pa (red line). (b) Evolution of the peak corresponding to free hydroxyl groups for different values of the cumulative strain γ_T_. Reprinted with permission under a Creative Commons Attribution Non-Commercial 3.0 Unported License from ref (78). Copyright 2019 Royal Society of Chemistry.

In terms of cellulosic materials, one of the main interaction parameters in solution, and in the solid state, is hydrogen bonding.^123,124^ The O–H vibrations are typically very prominent in IR spectra and can be used to track changes in the supramolecular structure of cellulose, as well as to indicate interactions with alien substances.^125−127^ In combination with rheometry, IR spectroscopy is capable of highlighting the failures of hydrogen-bonded networks upon shear. In the case of CNCs, this can be exploited to detect the failure of hydrogen bonds in a gel or in dispersions as a function of shear. One of the few accounts of CNC–polymer gel interactions used IR spectroscopy to prove that progressive and irreversible failure of the hydrogen bond network was progressing as shear rate increased (Figure 7).^78^ The authors of this study investigated two cases: one with cross-linking additives and one without. When no additives were present in the solutions, the polymer–CNC percolated network showed an immediate, partial recovery of the viscoelasticity thereof upon cessation of flow. In contrast, if additives were present, the recovery times of the gels were significantly longer, because the mechanism proceeded in accordance with van der Waal’s interactions. The authors of this study developed master curves that considered the temporal evolution of the viscoelasticity of polymer–CNC gels; these curves demonstrated that the observed dynamics can be generalized with respect to gel composition and flow conditions. Interestingly, they suggested that there were indications that polymer–CNC composite gels exhibit some distinctive features of colloidal glasses. The response of these gels to the flow conditions encountered during processing can be tailored with the inclusion of suitable additives.

**Figure 8:**
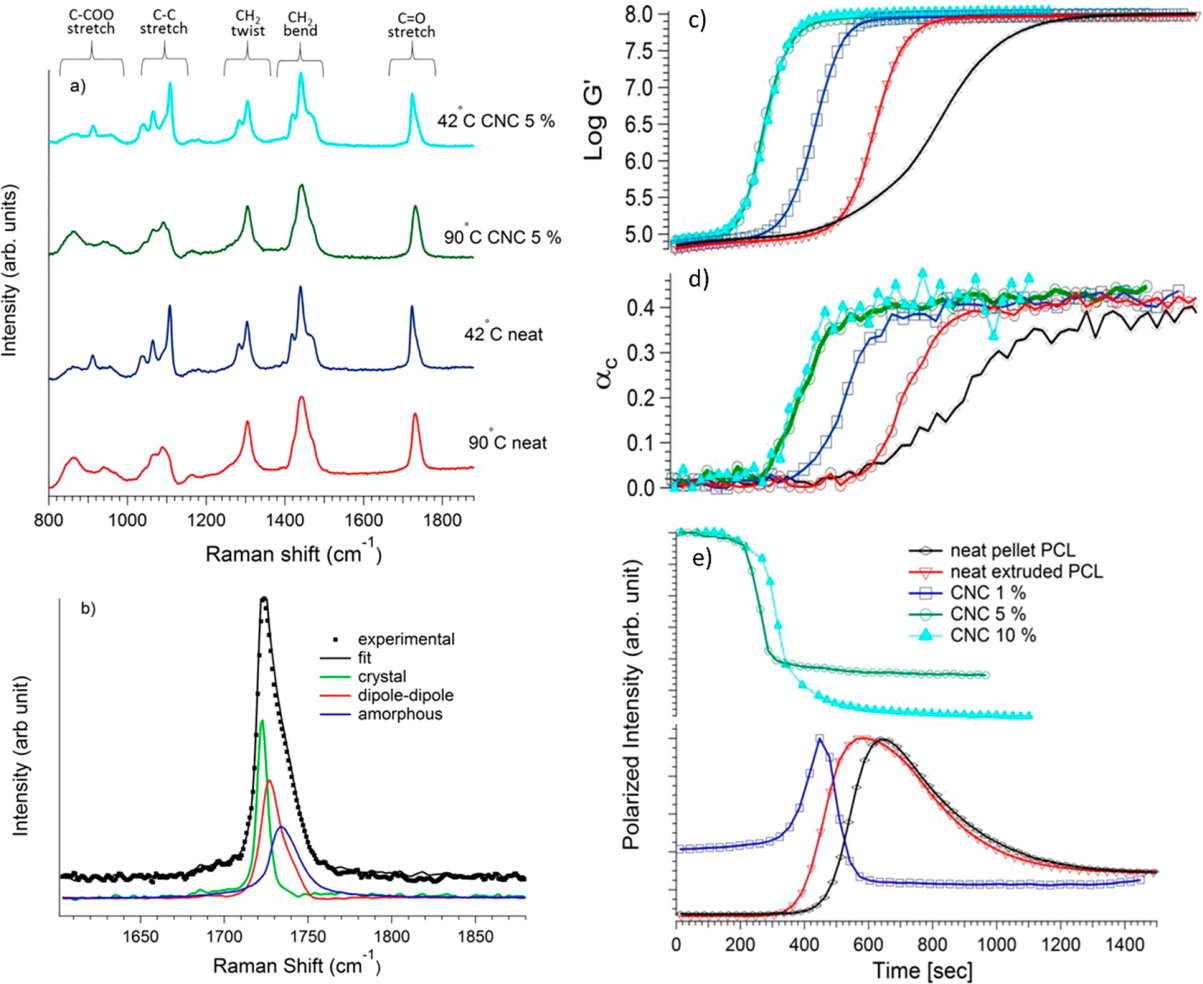
(a) Raman spectra of the 5% PCL/CNC nanocomposite (upper two curves) and the neat pellet PCL (lower two curves) in the melt (90 °C) and semicrystalline (42 °C) states. (b) Raman spectra in the C=O region showing the deconvolution of the curve peak into melt (amorphous and dipole–dipole) and crystal-based spectra at 1733 and 1722 cm^–1^, respectively. (c) Storage modulus, (d) Raman crystallinity, and (e) intensity profile for neat (pure and extruded) PCL and PCL/CNC (modified) composites performed at isothermal temperature, 42 °C. Reprinted with permission from ref (128). Copyright 2018 Elsevier.

Roy *et al*.^128^ investigated the cooling behavior of extruded melts containing poly-ε-caprolactone and sulfated CNCs. Even though the rheology of melts is very different from the core of this Review, it can showcase the potential of rheo-Raman for the identification of phase transitions in liquid crystalline states of CNC suspensions. Upon cooling, the Raman spectra of these melts were correlated to simultaneously acquired rheological data using a rheo-Raman device (Figure 8). Rheo-Raman eventually allows a clear understanding of the correlation between the growth of the transient mechanical modulus and that of crystallinity. In greater detail, the authors studied the effect of CNC concentration on isothermal crystallization kinetics. As processing with the sodium form of sulfated CNCs is challenging, they changed the cation to Bu_4_N^+^, followed by melt mixing *via* twin-screw extrusion. The crystallization rate increased as the CNC contents increased. Furthermore, a significant surge in the relative kinetics of an increase of the modulus versus that of crystallinity was observed with higher CNC concentrations (Figure 8c,d). The authors assessed the data using generalized effective medium theory and modeled the critical percolation thresholds. The result was a change in nucleation density in the anisotropy of crystallization.

### Current Thoughts on Opportunities and Challenges

Here, we will summarize the analytical possibilities of optics, scattering, and spectroscopy coupled to rheology. We will discuss the limitations, challenges, and potential opportunities in future endeavors with CNCs.

Coupling rheology to optics is relatively easy to implement that allows the visualization of features within the wavelength of visible light and reaches the 400–700 nm length-scales. In practice, an analysis with optical microscopy coupled to rheology has enabled visualization of regions of interest within a 100 μm scale bar, in addition to macroscopic features. Clearly, optical microscopy coupled to rheology does not reach individual particle length-scales; it can instead be used to study the assembly of particle clusters, but it can only indirectly access the liquid crystalline ordering *via* observation of the fingerprint pattern and orientation in the flow direction *via* the appearance of the Maltese-cross pattern. While the birefringence patterns in certain CNC suspensions contain vibrant coloration, basic qualitative observations cannot reveal the underlying structure of the polydomain texture. However, the predominant orientation of CNCs in the flow direction can be readily observed at the macroscale through the Maltese-cross pattern.

SAXS and neutron scattering are superior methods to analyze features that are approximately from one nanometer to 120 nm Analysis of smaller features is accessible with WAXS; however, these techniques are limited in a direct analysis of larger structures. Of course, larger structures can be accessed with scanning techniques, but an analysis of scanning techniques in flow can be limited by the acquisition times thereof. The 100 nm length-scale of interest for SAXS/SANS reveals its potential to answer questions about the evolution of the chiral nematic order in suspensions. Because assembly requires time, however, there is a practical challenge related to the measurement time, especially when it comes to utilization of synchrotron sources. However, the analysis does not cover macroscale aspects of the flow organization.

In chemistry, an NMR spectroscopic analysis is superior for resolving molecular structures. As expressed in the corresponding section, the relaxation effect can also be used to clarify the arrangement of particles in a suspension. The organized regions have a relaxation time that is different from the disorganized ones, and hence, NMR spectroscopy is available for such characterizations. While the high-resonance-frequency NMR spectrometers are still inflexible when it comes to coupling them to other analytics that require expensive inlets and adaptations, tabletop NMR spectrometers do not have this shortcoming. However, tabletop NMR spectrometers do not offer the option of gradient field pulses and have relatively low field strength, limiting their applicability for dynamic microscopy purposes. The determination of relaxation times is independent of the field strength, however, and can be used to observe motional differences between the different phases. Issues with resolution can be tackled by the more homogeneous magnetic fields of tabletop NMR spectrometers, and in the past few years, some suppliers (*e.g*., Bruker, X-Pulse, Oxford) have achieved major progress in this respect at field strengths of 1.4 T.

CNCs in NMR are challenging, because their particulate form impedes characterization by NMR spectroscopy due to rather short *T*_2_ times and concomitant line broadening. Reports have shown that differences in the diffusion behavior of different phases can be measured *via* the relaxation behavior of deuterated samples. Further developments in hardware and corresponding NMR experiments will contribute to a better picture of CNC phase behavior in aqueous suspensions and will clarify details of their formation and conversion into each other.

We foresee the following three open questions as topics for rheo-coupled techniques for CNC analysis. (i) Although alignment into the shear flow direction has already been characterized, the orientation and organization dynamics leading to unidirectional alignment still need to be better understood, especially in relation to the CNC phase and three-region behavior.^71,40^ (ii) Identification of the nanoscale orientation of individual crystals and the submicron hierarchies, especially the chiral nematic phase, still needs to be conducted. (iii) Recovery of the structures that are achieved in shear is another phenomenon that has proven to be challenging to comprehend; for example, the rheometer setup is typically limited to observations when no evaporation of the solvent has taken place. However, evaporation is part of the material processing and is a vital parameter to understand the production of CNC materials. It is also noteworthy that the scattering and spectroscopic techniques provide quantitative information. Crystallinity, sizes, and spacing of the features can be determined from the SAXS, SANS, and SALS and translated to degree of order. Spectroscopic analyses as well are able to resolve spatial information. The polarized light microscopy imaging is mostly qualitative. Furthermore, here we have focused on the hyphenated techniques for CNC structure characterization, but a similar toolbox can and has been used, *e.g*., for the analysis of crystallization in polymers in general.^129^ The techniques that have been reviewed are accessible as we have concentrated on commercial devices. However, there is a difference in costs of the infrastructure. Considering the costs of the hyphenated techniques, the PLI, basic FTIR, Raman, and tabletop NMR are more accessible than most of the scattering techniques. The neutron scattering accessibility is also steered by their availability solely at synchrotrons. The coupled data analyses also create a challenge related to the magnitude of data and data analysis; for example, while an analysis of scattering data requires expertise and computing, which is easily accomplished for static measurements, the multitude of available observations from both rheology and the coupled methods result in large amounts of data that need to be reduced and ultimately assessed and interpreted, which is a laborious task.

Although the main focus of hyphenated rheological setups applied to CNC suspensions has been the elucidation of shear-induced CNC phase behavior and structuring, it is worth noting that viscometric flows of suspensions can be prone to other phenomena that can induce inhomogeneities in flow, such as shear banding instabilities^130−132^ and wall slip.^133^ A general overview thereof with a focus on nanocellulosic systems can be found in Hubbe *et al*.^42^ In particular, apparent wall slip or wall depletion^133^ could occur especially in concentrated suspensions.^134,135^ Avoiding apparent slip is a significant challenge for rheo-PLI setups, where transparent glass surfaces and reflective metallic surfaces are required, thus prohibiting the use of geometries with roughened surfaces. This is not a limitation for scattering and spectroscopic combined rheological methods described in this Review. Combined rheology optical methods can, however, be used to assess apparent slip/wall depletion, as shown using the rheo-PLI setup with LS-echo in CNC suspensions^95^ or by optical coherence tomography in other nanocellulosic systems.^136^ In addition, rheometry combined with specialized scattering methods such as grazing-incidence SANS (GISANS) could be used to examine near-wall flow-induced phenomena.^137−139^ In principle, rheo-NMR in combination with pulsed field gradients or dynamic nuclear polarization could offer similar opportunities but requires still optimization in hardware design and setup.

A crucial aspect for the further development of the research and applications of CNCs is the development of standardized procedures for CNC preparation, CNC dispersion, and CNC analysis in the solid and dispersed state. Several efforts are underway in this respect, and it would be desirable to include rheology coupled with other methods as well in such a set of characterization techniques. This would allow for gaining better insights in the behavior of CNCs in the liquid crystalline state and may lead to a deeper understanding of the phase transitions and processes.

## Conclusion

The hierarchies of CNC suspensions require coupled analytics to reach nanometer (*i.e.*, individual particles), micrometer (*i.e.*, clusters of particles), and macroscopic (*i.e.*, the suspension) length-scales. Thus far, the coupled analyses have focused on revealing the alignment in shearing or the required shear conditions to attain unidirectional orientation. However, there are still unanswered questions regarding how the chiral order develops in suspensions and how it unravels in shear, and a comprehensive understanding of microphase transitions in suspensions is lacking. We foresee that the future of coupled techniques will need to expand to analyze the transitions that take place during the drying of structures after shearing, for example.

## Vocabulary

CNCs: Cellulose nanocrystals are 1D nanoparticles and part of the rodlike family of lyotropic materials.
PLI: A general term referring to optical imaging techniques involving polarized light. It has numerous applications across a broad variety of materials, including for the observation of liquid crystalline orders.
NMR: Nuclear magnetic resonance spectroscopy is an analytical method, capable to determine local fluctuations in a magnetic field. In chemistry, it is one of the most common methods to assess the chemical structure of molecules.
SAXS: Small-angle X-ray scattering is an X-ray-based method that allows for the investigation of nanoscale features of solid and dispersed materials. This includes the size distributions of different types of nanoparticles, pore sizes, and characteristic lengths (*e.g.*, in partially ordered structures) of materials.
SANS: Small-angle neutron scattering is an experimental technique similar to SAXS. The main difference is the use of neutrons as a radiation source which results in higher contrast, particularly for light elements.
